# The *Drosophila* MAST kinase Drop out is required to initiate membrane compartmentalisation during cellularisation and regulates dynein-based transport

**DOI:** 10.1242/dev.104711

**Published:** 2014-05

**Authors:** Daniel Hain, Alistair Langlands, Hannah C. Sonnenberg, Charlotte Bailey, Simon L. Bullock, H.-Arno J. Müller

**Affiliations:** 1 Division of Cell and Developmental Biology, College of Life Sciences, University of Dundee, Dundee DD1 5EH, UK; 2 Division of Cell Biology, MRC Laboratory of Molecular Biology, Francis Crick Avenue, Cambridge Biomedical Campus, Cambridge CB2 0QH, UK

**Keywords:** *Drosophila*, Protein kinase, Cell polarity, Microtubules, Dynein

## Abstract

Cellularisation of the *Drosophila* syncytial blastoderm embryo into the polarised blastoderm epithelium provides an excellent model with which to determine how cortical plasma membrane asymmetry is generated during development. Many components of the molecular machinery driving cellularisation have been identified, but cell signalling events acting at the onset of membrane asymmetry are poorly understood. Here we show that mutations in *drop out* (*dop*) disturb the segregation of membrane cortical compartments and the clustering of E-cadherin into basal adherens junctions in early cellularisation. *dop* is required for normal furrow formation and controls the tight localisation of furrow canal proteins and the formation of F-actin foci at the incipient furrows. We show that *dop* encodes the single *Drosophila* homologue of microtubule-associated Ser/Thr (MAST) kinases. *dop* interacts genetically with components of the dynein/dynactin complex and promotes dynein-dependent transport in the embryo. Loss of *dop* function reduces phosphorylation of Dynein intermediate chain, suggesting that *dop* is involved in regulating cytoplasmic dynein activity through direct or indirect mechanisms. These data suggest that Dop impinges upon the initiation of furrow formation through developmental regulation of cytoplasmic dynein.

## INTRODUCTION

Primary epithelium formation during embryonic cleavage divisions provides an excellent model with which to unravel the mechanisms that initiate the segregation of the plasma membrane cortex into distinct subdomains (Müller, 2001). In *Drosophila* this process occurs during cellularisation and comprises successive subcellular events that culminate in the formation of the monolayered, polarised blastoderm epithelium (Lecuit and Wieschaus, 2000; Mazumdar and Mazumdar, 2002; Lecuit, 2004). Cortical asymmetry becomes evident by accumulation of proteins including F-actin and Myosin II, which mark the sites where cleavage furrows invaginate (Sokac and Wieschaus, 2008b). These incipient cleavage furrows mature to form furrow canals that are stabilised by actin/myosin, the actin regulators RhoGEF2, Rho1 and Diaphanous (Dia), and additional regulatory proteins including Slow as Molasses (Slam), Septins, Nullo and Patj (Hunter and Wieschaus, 2000; Lecuit et al., 2002; Grosshans et al., 2005; Sokac and Wieschaus, 2008b; Wenzl et al., 2010). Basal adherens junctions (bAJs) form apical to the furrow canal and move inward with the furrows (Müller and Wieschaus, 1996; Hunter and Wieschaus, 2000). As furrows move basally, spot adherens junctions (sAJs) form along the cell interface of the newly forming membranes (Tepass and Hartenstein, 1994; Müller and Wieschaus, 1996). The assembly of sAJs into apical adherens junctions (aAJs) is controlled by Bazooka (Baz), a scaffold protein localised in the apical domain (Müller and Wieschaus, 1996; Harris and Peifer, 2004). How Baz is initially localised apically is not understood, but both an actin-based cytoskeletal scaffold and dynein-dependent microtubule-based transport play important roles in this process (Harris and Peifer, 2005).

Formation of the furrow canal is tightly linked to membrane turnover and membrane growth. The fusion of endomembrane pools with the outer cell membrane, as well as endocytosis, is important for furrow canal morphogenesis (Lecuit and Wieschaus, 2000; Pelissier et al., 2003; Sokac and Wieschaus, 2008a). Membrane growth requires an intact microtubule cytoskeleton and vesicle trafficking through the secretory pathway and the recycling endosome (Lecuit and Wieschaus, 2000; Sisson et al., 2000; Pelissier et al., 2003). Compromising the function of cytoplasmic dynein blocks furrow progression, suggesting that transport along microtubules by this minus end-directed motor plays an important role in membrane growth (Papoulas et al., 2005).

Here we show that mutations in *drop out* (*dop*) disrupt the formation of incipient furrow canals. *dop* encodes the single fly homologue of microtubule-associated Ser/Thr (MAST) kinases, a poorly characterised, but highly conserved subfamily of AGC (PKA, PKG, PKC) kinases (Pearce et al., 2010). The founding member, mammalian MAST2, was originally found to co-fractionate with microtubules and to bind and control phosphorylation of the tumour suppressor PTEN and the Na^+^/H^+^ exchanger NHE3 (SLC9A3) (Walden and Cowan, 1993; Valiente et al., 2005; Wang et al., 2006). Only a small number of additional molecular interactions of MAST kinases have been reported; these include interactions with Traf6, PCLKC and β2-synthrophin (Lumeng et al., 1999; Okazaki et al., 2002; Xiong et al., 2004). Despite the association of MAST kinases with diseases including inflammatory bowel disease and breast cancer (Labbé et al., 2008; Robinson et al., 2011), the physiological functions of this protein family within the developing or adult organism remain elusive. Our findings show that Dop plays a crucial role in dynein-dependent microtubule-based transport and is required for the phosphorylation of Dynein intermediate chain. We propose that regulation of dynein-dependent transport by Dop is important during the early steps of generating cortical plasma membrane asymmetries in the course of embryonic epithelium formation.

## RESULTS

### *dop* encodes the single fly homologue of MAST kinases

**Fig. 1.**
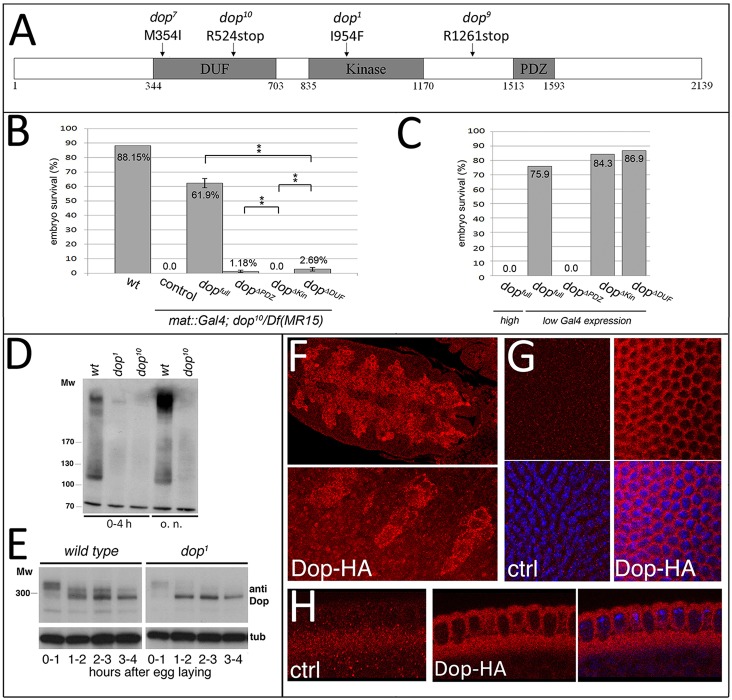
**Characterisation of *Drosophila dop* mutations and protein.** (A) Location of mutations in EMS-induced *dop* mutant alleles in *CG6498*. Conserved protein domains are in grey; numbers indicate amino acids. (B) Genetic complementation of *dop* mutants. Embryos were derived from *dop^10^/Df(3L)MR15* females expressing Dop-HA and constructs lacking the DUF, kinase or PDZ domain using *mat67*-Gal4. **P<0.005 (*t*-test); error bars indicate s.d. [*n*=512, 566, 631, 1296, 747, 1130 (from left to right)]. (C) Effect on embryo survival of overexpression of Dop and constructs in B using maternal drivers at high (*mat-67-*Gal4*;mat-15-*Gal4) or low (*nos-*Gal4) expression. (D) Immunoblot analysis of embryo lysates using anti-Dop antibody. Embryo collections were either 0-4 h or overnight (o.n.). (E) Anti-Dop immunoblot of lysates from embryos of the indicated ages. α-Tubulin (tub) provides a loading control. (F) Anti-HA immunolabelling of Dop-HA expressed with *twi-*Gal4 in the mesoderm. Lower panel is a higher magnification of mesoderm cells. (G,H) Anti-HA staining of slow phase stage embryos obtained from *w^1118^* (ctrl) or *mat67-*Gal4/UAS-*Dop-HA;dop^10^/Df(3R)MR15* females. (G) Surface view: top, anti-HA; bottom, merge (HA, red; DAPI, blue). (H) Transverse sections: left and middle, anti-HA; right, merge (HA, red; DAPI, blue). See also supplementary material Fig. S2 and Movies 1 and 2.

*dop^1^* was originally described as a recessive female sterile mutation, but its molecular nature remained unclear (Galewsky and Schulz, 1992). Although the *dop^1^* mutant can be rescued to some extent by expression of the RNA-induced silencing complex component *Argonaute 2* (*Ago2*) (Meyer et al., 2006), detailed analysis revealed that *dop^1^* does not represent an allele of *Ago2* (Hain et al., 2010). We used the original *dop^1^* allele to identify novel non-complementary mutations generated by chemical mutagenesis. Following genetic mapping (supplementary material Fig. S1A), genomic DNA sequencing revealed mutations in *CG6498*, which encodes the single *Drosophila* homologue of the MAST kinase family (Fig. 1A; supplementary material Fig. S1). MAST kinases share a conserved domain structure, with a central AGC kinase domain flanked by a Pfam DUF1908 (conserved in all known MAST kinases) and a PDZ (PSD95, Dlg, ZO-1) domain (Fig. 1A; supplementary material Fig. S1B). The *dop^1^* allele contains a missense mutation of a conserved isoleucine 954 within the kinase domain. In the case of RSK2 (RPS6KA3), another member of the AGC kinase family, mutation of this residue in humans with X-linked mental retardations is associated with reduced kinase activity (Delaunoy et al., 2001). These data suggest that *dop^1^* represents a loss-of-function allele.

Like *dop^1^*, all of the new *dop* alleles were female sterile, embryonic lethal and produced membrane growth defects in cellularisation. In addition, homo- or hemizygous adults had variable wing and leg defects, suggesting a role of *dop* in imaginal disc development (see below; data not shown). The *dop^10^* allele is predicted to encode a severely truncated protein; the *dop^9^* and *dop^7^* alleles suggest important roles for the PDZ domain and the DUF domain, respectively (Fig. 1A). Maternal expression of *CG6498* using the binary *Gal4-UAS* system resulted in rescue of the lethality of *dop* mutations in over 60% of mutant embryos (Fig. 1B). The incomplete rescue is likely to be a result of inappropriate expression levels as a consequence of using the *Gal4* system. Indeed, overexpression of *CG6498* in wild type caused lethality and early embryonic cleavage arrest (Fig. 1C; data not shown). These effects were dependent on the strength of *Gal4* expression and on the presence of the PDZ domain, suggesting that overall Dop protein level and the PDZ domain are crucial in the regulation of Dop (Fig. 1C). Expression of constructs lacking either the PDZ or DUF domains rescued at a low level, whereas a kinase-deficient protein did not rescue (Fig. 1B). Taken together, we conclude that *dop* is allelic to *CG6498* and that the PDZ and DUF domains provide important accessory functions, while the kinase domain is essential.

Antibodies against Dop detected a smear of bands on immunoblots of wild-type embryo lysate with an apparent molecular weight between 350 kDa and 280 kDa (Fig. 1D). These bands were strongly reduced or absent in lysates from *dop^1^* or *dop^10^* mutants, respectively (Fig. 1D). Dop protein has a calculated molecular weight of ∼225 kDa, suggesting that Dop is post-translationally modified. The genome annotation for *CG6498* predicts two differentially spliced transcripts that only differ in their 3′ untranslated region (UTR), rendering the presence of higher molecular weight protein isoforms unlikely. On immunoblots Dop protein exhibited interesting dynamics in early embryos. The higher molecular weight form (>300 kDa) prevailed in early cleavage stages (Fig. 1E). This predominant form was progressively shifted to a lower molecular weight form (<300 kDa) in syncytial stages and during cellularisation. In *dop^1^* mutants the higher molecular weight forms were strongly reduced throughout early development (Fig. 1E). Since *dop^1^* affects the kinase domain, it is conceivable that the post-translational modification of Dop depends on its kinase activity. These data demonstrate that Dop is post-translationally modified in a developmentally controlled fashion.

Antibodies against Dop did not reveal specific staining in embryos (D.H. and H.-A.J.M., unpublished). Tissue-specific expression of a transgenic HA-tagged Dop protein in embryos revealed that the protein is distributed throughout the cytoplasm and absent from nuclei (Fig. 1F). Likewise, in cellularisation stage embryos, HA-tagged Dop protein was present throughout the cytoplasm (Fig. 1G,H). GFP-Dop protein did not exhibit obvious dynamic movement during cellularisation, but during syncytial stages it was dynamically associated with the mitotic spindle or associated structures (supplementary material Fig. S2 and Movies 1 and 2). Since *dop* mutants showed no defects in syncytial divisions, we did not investigate the nature of this dynamic behaviour of Dop-GFP. We conclude that Dop is a cytoplasmic protein kinase that is developmentally regulated during blastoderm development.

### Dop is required for cortical plasma membrane asymmetry

**Fig. 2.**
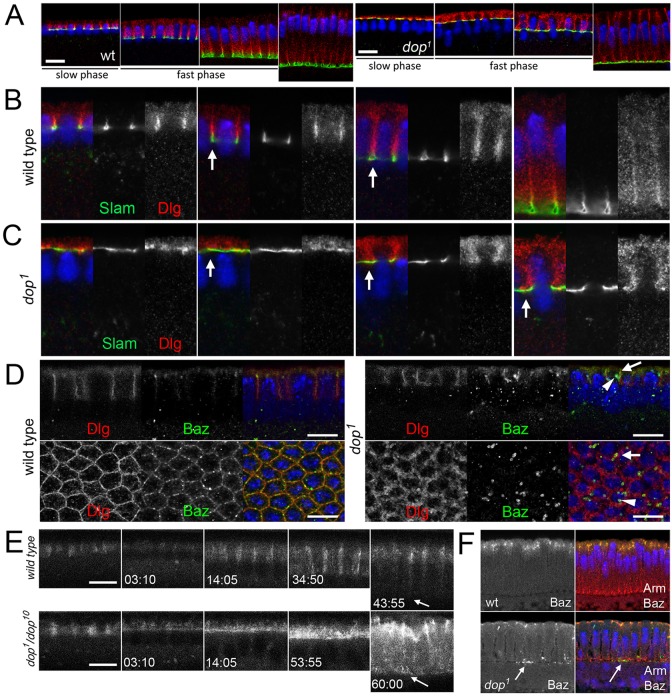
**Defects in membrane compartmentalisation in *dop* mutants.** (A) Overview of progressive cellularisation (left to right) from slow phase through fast phase in fixed wild-type and *dop^1^* embryos. Slam, green; Dlg, red; DNA, blue. Staging was according to nuclear elongation. (B,C) Magnifications of A (panel width represents 10 µm). Note the abnormally wide furrows (arrows in C) in mutants compared with wild type (arrows in B). (D) Wild-type and *dop^1^* embryos stained for Dlg and Baz (top, transverse view; bottom, surface view). Arrows point to Baz puncta colocalising with Dlg and arrowheads point to Baz puncta that do not colocalise with Dlg. (E) Transverse optical sections showing Baz-GFP movement in the lateral membrane. Baz-GFP is frequently observed to remain within the basal cytoplasm (arrows). (F) Endogenous Baz in fixed embryos also remains in the basal cytoplasm (arrow). Arm, red; Baz, green; DAPI, blue. Time is indicated in min:s. Scale bars: 10 µm.

The identification of *dop* as the single fly homologue of the MAST kinases offers an excellent opportunity to gain insight into the function of this highly conserved and biomedically relevant branch of AGC kinases. Previously, we showed that females homozygous for *dop* mutant alleles produce embryos that exhibit severely delayed membrane growth during cellularisation (Meyer et al., 2006). During cellularisation, the membrane cortex undergoes a successive compartmentalisation into distinct subdomains: apical membrane, furrow canal, bAJ, lateral membrane and aAJ (Müller and Bossinger, 2003). In normal development, the apical protein Baz, the lateral protein Discs large (Dlg, also known as Dlg1) and the furrow canal marker Slam become separated into distinct domains. Slam resides at the furrow canal that forms as a discrete and continuous folding of the plasma membrane at an equidistant interface between the nuclei (Fig. 2). Dlg is found at the lateral membrane and becomes excluded from the furrow canal as cellularisation progresses (Fig. 2A,B). In *dop* mutant embryos, the segregation of these membrane-associated proteins is delayed and incomplete. During early stages of cellularisation, Slam was mislocalised to an expanded cortical compartment and failed to localise into discrete foci (Fig. 2B,C). At mid- to late cellularisation, malformed furrows did move basally, confirming our earlier data showing that membrane growth was strongly delayed in *dop* mutants (Meyer et al., 2006). Dlg protein localisation extended into the Slam domain even at mid- and late cellularisation (Fig. 2A,C; supplementary material Fig. S3).

We next examined the distribution of the apical protein Baz. In mid- to late cellularisation, Baz mostly overlapped with Dlg in the apical-lateral membrane domain (Fig. 2D). In *dop* mutants, the bulk of Baz protein was localised to abnormal aggregates, which were often localised within the cytoplasm and partially colocalised with cortical Dlg protein (Fig. 2D). Baz protein moves apically into the region that will define the aAJ (Harris and Peifer, 2005) and was depleted from the basal cytoplasm after 45 min into cycle 14 (Fig. 2E; supplementary material Movie 3). In *dop* mutant embryos, Baz-GFP did show some apical movement, but basal depletion was incomplete; basal Baz-GFP or endogenous Baz was still found at the onset of gastrulation (Fig. 2E,F; supplementary material Movie 3 and Fig. S4). We conclude that mutations in *dop* affect the segregation of apical and basal plasma membrane compartments throughout cellularisation. Abnormal localisation of Baz and reduced membrane growth both point to defects in the transport and sorting of integral and associated membrane proteins in the absence of *dop*.

### *dop* mutations affect formation of the furrow canal and the basal junction

**Fig. 3.**
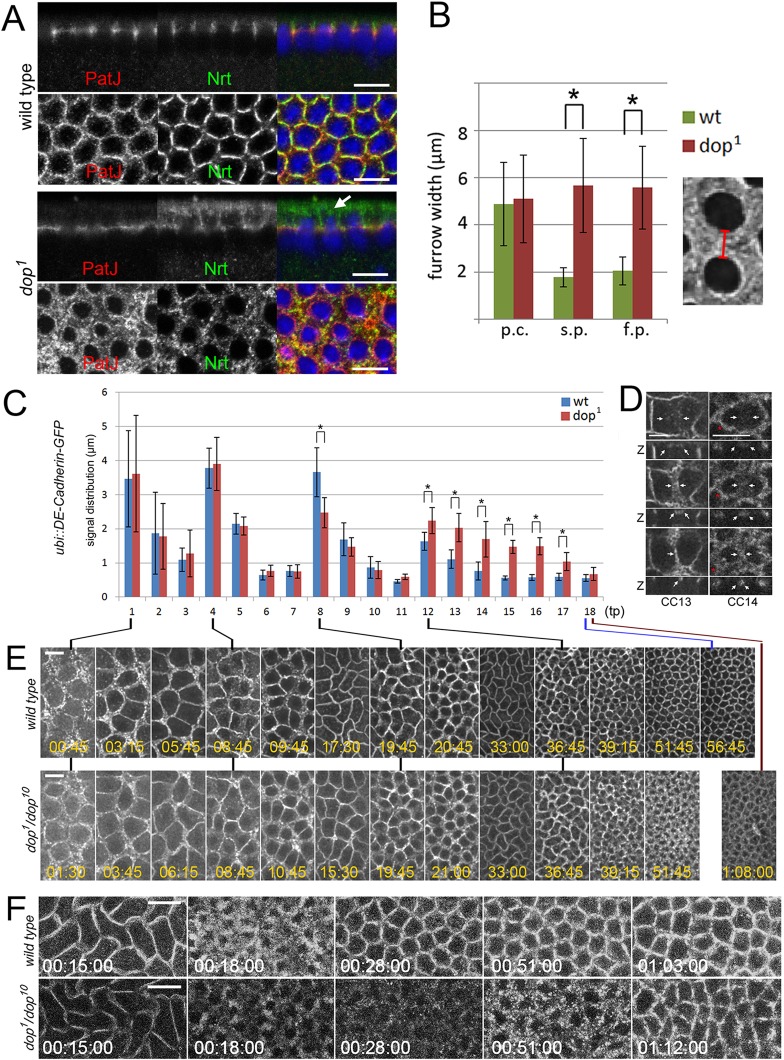
**Furrow and bAJ formation in *dop* mutants.** (A) Wild-type and *dop^1^* embryos stained for Patj and Neurotactin (Nrt) showing abnormal furrow canals and apical aggregates of Nrt (arrow). The lower panel of each pair shows a focal plane on the level of furrows. (B) Measurement of furrow width (see inset) indicated by Slam localisation in pre-cellularisation (p.c.), slow phase (s.p.) and fast phase (f.p.). *P<0.0001 (*t*-test); error bars indicate s.d. (*n*>230 for each sample). (C-E) Live imaging of E-cadherin-GFP in wild-type and *dop^1^* mutant embryos in syncytial and cellularisation stages. (C) Focussing of E-cadherin-GFP into the basal junctions during cell cycles 11-14. The extent of E-cadherin-GFP was measured across the newly forming furrow (see D). *P<0.001 (*t*-test); error bars indicate s.d. (*n*=25 furrows over time of two distinct embryos each). (D) E-cadherin-GFP dynamics in cell cycle (CC) 13 and 14 in *dop^1^* embryos. Z marks resliced stacks showing the furrow region. Arrows mark the edge of cell cycle 13 E-cadherin-GFP clusters moving towards clearly demarcated junctions; note that at the cell cycle 14 transition, not only do junctions not focus at new furrows but also previously existing junctions become diffuse (red asterisks). (E) E-cadherin-GFP during cell cycles 11-14. (F) Baz-GFP at the cell cycle 13-14 transition in wild type and *dop^1^*. Time is indicated in h:min:s. Scale bars: 10 µm.

The defects in membrane growth and cortical membrane compartmentalisation might be consequences of a malformation of the furrow canal and the bAJ. Both of these structures form during the first 10 min of interphase in cycle 14. Similar to Slam, the furrow canal protein Patj rapidly accumulated in sharp, regular foci equidistant from the nuclei; however, in *dop* mutants Patj protein localisation remained unfocussed and irregular during this early phase (Fig. 3A). To determine whether these defects are a consequence of earlier defects during syncytial development, we measured the width of the furrows using Patj as a marker. Furrow widths were unimpaired in syncytial development, but during cellularisation they were significantly wider and irregular in *dop* mutants compared with controls (Fig. 3B). Thus, Dop is dispensable for metaphase furrows but important for normal furrow formation in cellularisation.

Given this phenotype, Dop might function at the transition from syncytial cleavages to cellularisation. During this transition, E-cadherin (also known as Shotgun) becomes redistributed from apical villous projections to bAJs (McGill et al., 2009). Before cellularisation, E-cadherin-GFP accumulated at sharp boundaries between dividing nuclei in both wild type and *dop* mutants (Fig. 3C,E). However, at the cycle 14 transition, E-cadherin-GFP failed to focus into the furrow region in *dop* mutants and E-cadherin-GFP puncta persisted in the apical region (Fig. 3C-E; supplementary material Movies 4 and 5).

Baz plays a major role in the assembly of E-cadherin puncta into larger precursors of AJs (McGill et al., 2009). During cellularisation, Baz-GFP exhibits a dynamic behaviour that eventually results in its apical accumulation (Harris and Peifer, 2005). Early in cellularisation, Baz interacts with puncta of E-cadherin at apical, actin-rich villous membrane projections to enable the enrichment of these proteins into the premature bAJ (McGill et al., 2009). In syncytial stages, Baz-GFP was localised to the metaphase furrows that surround the dividing chromosomes (Fig. 3F). At the cycle 14 transition, Baz-GFP localisation at the future apical cell interfaces became diffuse and then sharpened into clear-cut borders at future apical cell interfaces after ∼10 min into cycle 14 (Fig. 3F; supplementary material Movie 6). In *dop* mutants, Baz-GFP localised normally to metaphase furrows, but in cellularisation Baz-GFP remained diffuse and did not focus at cell interfaces until ∼50 min after the onset of cycle 14 (Fig. 3F; supplementary material Movie 6). Thus, Dop is required at the cycle 14 transition for the apical redistribution and focussing of both E-cadherin and its regulator Baz.

### Dop acts upstream of F-actin in incipient furrow formation

**Fig. 4.**
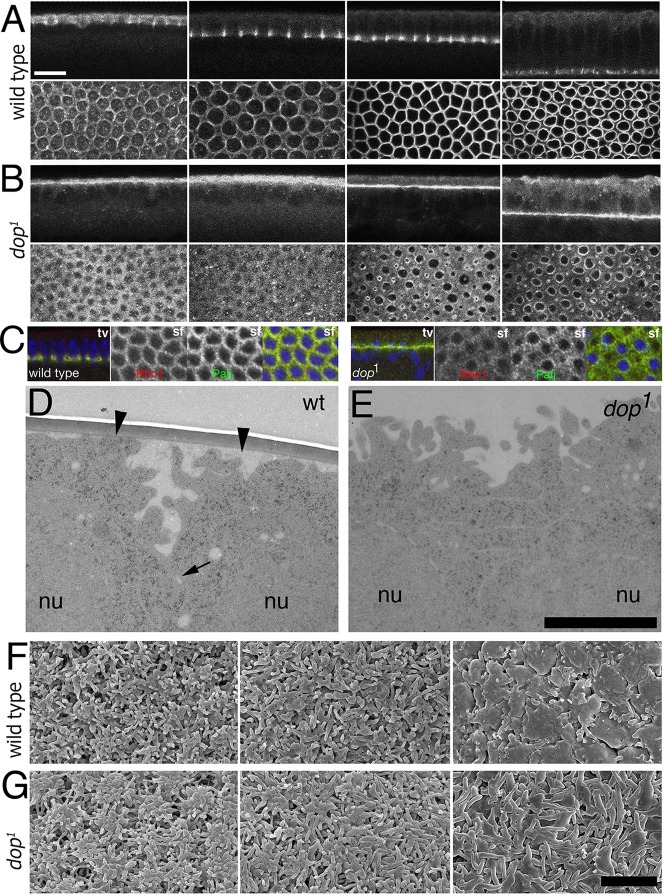
**Distribution of F**-**actin and furrow formation in *dop* mutants.** (A,B) Rhodamine-Phalloidin staining of wild-type (A) and *dop^1^* (B) embryos. Images from left to right show progressive stages of cellularisation. Upper rows show a transverse optical section and lower rows show a focal plane on the level of the furrows. (C) Wild-type and *dop^1^* embryos stained for Rho1 (red) and Patj (green). tv, transverse optical section; sf, surface optical section; colour images represent merged channels. (D,E) Transmission electron micrograph at the interface of two adjacent emerging cells (arrowheads) during slow phase. Note that the furrow canal extension (arrow) in the wild type is missing in the *dop^1^* mutant. nu, nucleus. (F,G) Scanning electron micrographs showing villous projections on the surface of wild type (F) and *dop^1^* mutant (G) at different time points during cellularisation. Left, cycle 14 transition; middle, slow phase; right, fast phase. Scale bars: 10 µm in A,B; 2.5 µm in D,E; 5 µm in F,G.

The defects in initial Baz redistribution and bAJ formation might be explained by an earlier defect in the generation of the incipient furrows. Within the first 5 min of cellularisation the formation of incipient furrows is marked by a shift of F-actin to the future cell interfaces (Warn and Magrath, 1983). In wild type, F-actin redistributes from an even cortical distribution to mark the incipient furrows at equidistant positions between the nuclei (Fig. 4A). As furrows moved basally, F-actin became focused to the furrow tips and was reduced in the apical cortex (Fig. 4A). In *dop* mutants, F-actin remained diffuse within the apical cortex from the early stages to mid-cellularisation (Fig. 4B). Despite these dramatic defects in F-actin relocalisation, the organisation of the microtubule cytoskeleton appeared largely unimpaired in *dop* mutants (supplementary material Fig. S5). We conclude that *dop* is required for the initial redistribution of F-actin to the sites of incipient furrows. Rho1, an important regulator of furrow formation and actin polymerisation at the furrows (Grosshans et al., 2005), exhibited a highly abnormal distribution that was similar to the abnormal localisation of F-actin (Fig. 4C). Since Rho1 activation relies upon the localisation of RhoGEF2 via Slam, mislocalisation of Rho1 might be a result of abnormal Slam localisation, rather than a cause. Thus, abnormal Slam localisation might contribute to a sustained failure in actin deposition in mid- and late cellularisation stages.

The maintenance of a uniform arrangement of cortical F-actin suggested that the initiation of furrow formation was blocked in *dop* mutants. Using transmission electron microscopy we found that furrow canals were indeed absent in *dop* mutants during the first 20 min after the onset of cycle 14 (Fig. 4D,E). Instead of areas of smooth membrane where furrow canals projected towards the basal cytoplasm, in *dop* mutants villous projections were prominent throughout and no furrow canals were present (Fig. 4E). As F-actin is also present in villous projections at the surface, we examined whether lack of *dop* has any impact on these protrusions (Turner and Mahowald, 1976). In *dop* mutants, large villous projections are present throughout cellularisation (Fig. 4F,G). In early cellularisation, there was no detectable difference in the distribution and size of the villous projections; however, towards the end of cellularisation, long projections persisted in *dop* mutants, whereas the surface became smooth in the wild type (Fig. 4F,G). Thus, mutations in *dop* did not generally affect F-actin-dependent structures during slow phase, suggesting that Dop is an essential factor that controls the redistribution of F-actin at the cycle 14 transition.

### *dop* mutations synergise with mutations of the dynein/dynactin complex

The phenotypes of *dop* mutants are reminiscent of the requirement for microtubules in cellularisation. Inhibitor studies have indicated that microtubules are essential for membrane growth in early but not late stages of cellularisation (Foe et al., 1993; Lecuit and Wieschaus, 2000). We previously reported that *dop* is required for the dynein-based apical transport of lipid droplets (Gross et al., 2000; Meyer et al., 2006). To further address the correlation between *dop* and dynein we examined genetic interactions between *dop* and genes encoding components of the dynein/dynactin complex.

**Fig. 5.**
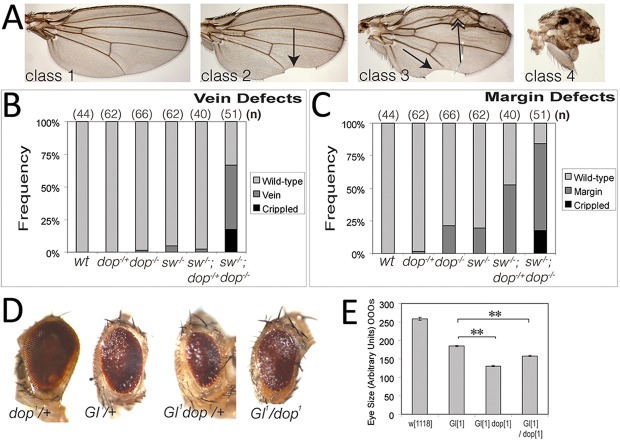
**Genetic interactions of *dop* with the dynein/dynactin complex.** (A) *dop^1^* wing phenotypes are enhanced by *sw^1^*. Four different classes of wing phenotypes occurring in adults mutant for *dop* or *sw*: wild type (class 1), margin defects (class 2, arrow), vein defects (class 3, double-headed arrow), or crippled (class 4). (B,C) Quantification of the occurrence of these phenotypic classes for the indicated genotypes. (D,E) Eyes from single- and double-mutant flies carrying *dop^1^* and *Gl^1^* alleles were imaged and the eye size represented in arbitrary units. (E) *dop^1^* enhances the *Gl^1^* eye phenotype in a dominant fashion: for *Gl^1^,dop^1^* compared with *Gl^1^* control, *P*=1.629×10^–63^; for *Gl^1^/dop^1^* compared with *Gl^1^* control, *P*=9.502×10^–12^; **P<0.001 (*t*-test); error bars indicate s.e.m.; *n*>80 for all genotypes.

Hypomorphic mutations in *short wing* (*sw*), which encodes *Drosophila* Dynein intermediate chain (Dic), exhibit similar adult wing phenotypes to homozygous *dop* mutants; the wing margins, in particular at the posterior, were partially deleted and wing veins were also affected (Fig. 5A) (Boylan and Hays, 2002). In *sw^1^ dop^1^* double mutants, the wing phenotype was enhanced, suggesting that the two genes affect a common process (Fig. 5B,C). Given the positive interaction of *dop* and *sw*, we investigated whether *dop* interacts with the dynactin component Glued (also known as p150/Glued). Glued binds Dic and the central dynactin component Arp1 and thereby allows dynein to connect to cargo and enhances processivity (Schroer, 2004). The dominant-negative mutant *Glued^1^* (*Gl^1^*) encodes a protein that can still bind to dynein, but has lost its ability to bind to the dynactin complex (McGrail et al., 1995). *Gl^1^* induces a rough eye phenotype in flies due to effects on a range of cellular processes, including nuclear localisation (Fan and Ready, 1997). The *Gl^1^* eye phenotype is sensitive to the doses of *sw* and we find that this phenotype is also dominantly enhanced by *dop^1^*, which on its own does not exhibit a rough eye phenotype (Fig. 5D,E). We conclude that *dop* function is not restricted to cellularisation and that dynein/dynactin-dependent processes are sensitive to the levels of Dop.

### Dop is required for phosphorylation of Dic

**Fig. 6.**
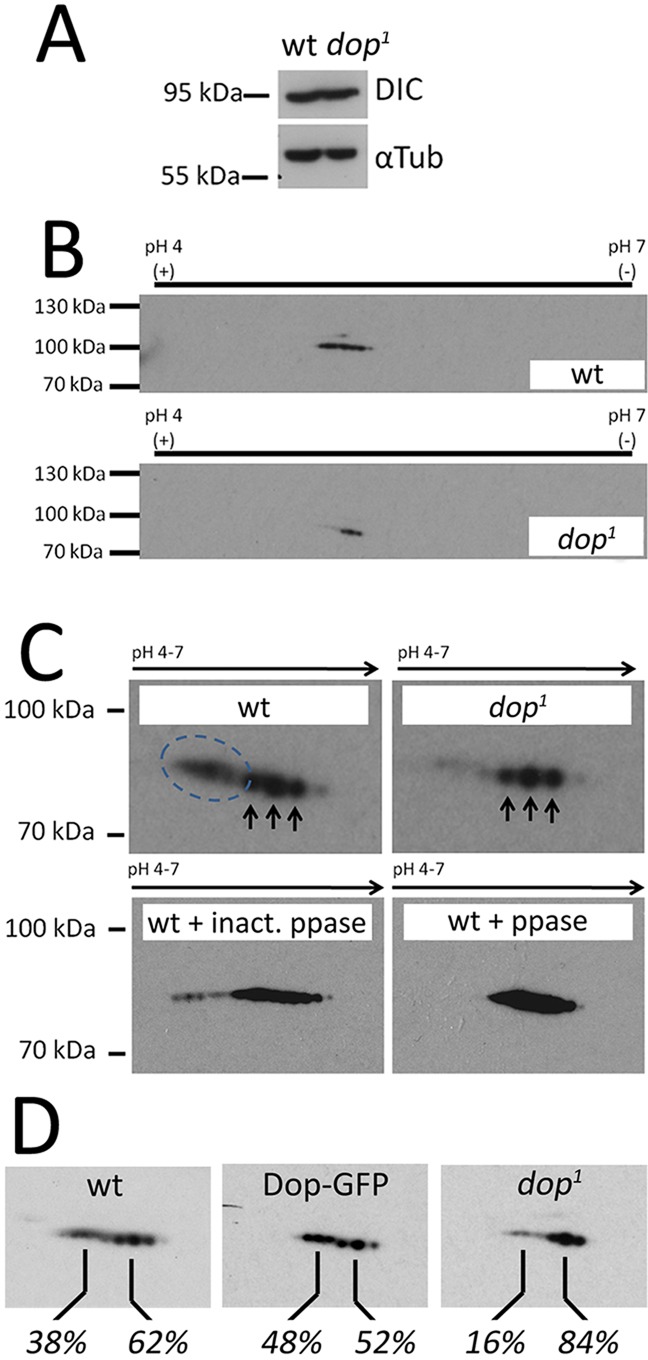
**Dop is crucial for phosphorylation of Dic.** (A) Protein extracts of wild-type and *dop^1^*embryos (at 0-4 h) were used for immunoblotting using anti-Dic antibody. α-Tubulin was used as loading control. (B-D) Separation of Dic by 2D gel electrophoresis. Black bars indicate the axis of the first dimension. (C) Magnification of the Dic 2D electrophoresis migration pattern. Spots of Dic isoforms are marked by arrows. A row of protein spots is detected in wild type (dashed circle) that is reduced in *dop^1^* mutant protein extract. These acidic spots are sensitive to treatment with active (+ ppase) but not inactivated (+ inact. ppase) phosphatase. (D) Overexpression of Dop-GFP increases Dic phosphorylation. Genotypes: wt is Gal4 driver alone (*mat67::Gal4;mat15::Gal4*); Dop-GFP is *mat67::Gal4;mat15::Gal4/UAS::Dop-GFP*; *dop^1^* is *dop^1^/dop^1^*. The percentages of the relative intensities of phospho-Dic (left number for each panel) and non-phospho Dic (right number for each panel) are indicated.

Since the function of Dop relies upon its protein kinase activity, the simplest model of how Dop regulates dynein-dependent transport would be by controlling the phosphorylation of the dynein/dynactin complex. Phosphorylation of Dic at multiple sites regulates the interaction of dynein with Glued and controls persistent minus end-directed transport (Vaughan and Vallee, 1995; Vaughan et al., 2001; Whyte et al., 2008; Ikeda et al., 2011). We examined Dic phosphorylation in *dop^1^* embryos. On immunoblots, the levels of Dic are unimpaired in *dop* mutants compared with wild type (Fig. 6A). After 2D gel electrophoresis, Dic isoforms were detected as a number of closely associated spots (Fig. 6B). These data are consistent with previous observations on *Drosophila* Dic demonstrating at least ten differentially spliced isoforms (Nurminsky et al., 1998). Some of these spots were at a slightly higher apparent molecular weight in the acidic region and were sensitive to phosphatase treatment, indicating that they represent phosphorylated forms (Fig. 6B). In extracts of *dop^1^* mutant embryos, phosphorylated forms of Dic are strongly reduced, whereas overexpression of Dop in the embryo increases the amount of phosphorylated Dic (Fig. 6B-D). We conclude that Dop either directly or indirectly controls phosphorylation levels of Dic, suggesting that Dop is involved in the regulation of dynein-based transport in the embryo.

### Dop is required for dynein-based mRNA transport in the embryo

The genetic interaction studies suggested that Dop might have a more general role in dynein-mediated processes. To test this notion, we examined the apical transport of mRNAs in *dop* mutant embryos. Apical mRNA movement in the embryo is a well-characterised system that depends on minus end-directed transport by dynein and dynactin (Wilkie and Davis, 2001; Bullock et al., 2006). One advantage of this system is that the motion of mRNA particles can be characterised in detail by microinjection of highly fluorescent RNAs coupled to rapid time-lapse imaging (Bullock et al., 2006). Similar to lipid droplets, transport of mRNA particles along microtubules occurs in a bi-directional fashion and net transport can occur as a result of changes in the frequency, the persistence (run length) and the velocity of plus end- versus minus end-directed bouts of transport (Bullock et al., 2006; Vendra et al., 2007).

**Fig. 7.**
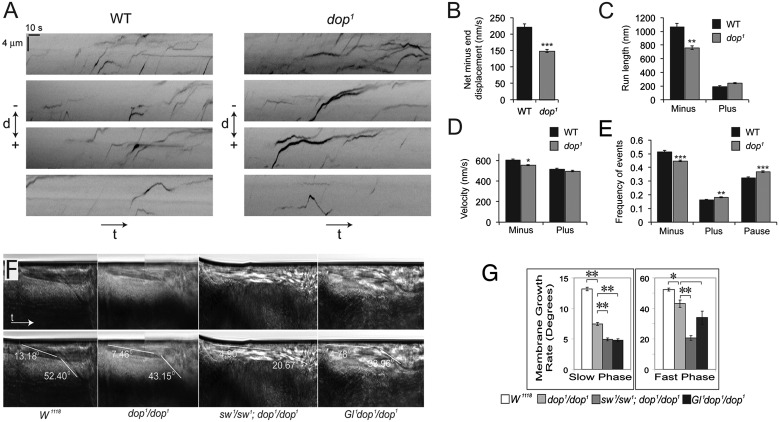
**Dop is required for dynein-based apical transport of *h* transcripts.** Alexa488-labelled RNA corresponding to the *h* 3′UTR was injected into cellularising embryos before the fast phase of cellularisation. The movement of fluorescent particles was recorded by video microscopy (supplementary material Movies 7 and 8). (A) Kymographs showing movement of a non-biased selection of Alexa488-labelled RNA particles. Four examples are shown for wild-type and *dop^1^* embryos (t, time; d, distance in minus end/plus end polarity). Particles have an overall minus end-directed bias in both genotypes. (B-E) Quantification of RNA motility resulting from automatic tracking and analysis of a larger number of RNA particle trajectories. (B) Mean rate of net minus end displacement per RNA particle. (C) Mean length per RNA particle of runs towards the minus or plus end. (D) Mean velocity per particle in plus or minus end direction. (E) Mean frequency per RNA particle of minus end- versus plus-end-directed motion and pauses. *P<0.05, **P<0.01, ***P<0.001 compared with wild type (ANOVA test with a nested unbalanced model); error bars indicate s.e.m. (F) Kymographs showing membrane growth in wild type, *dop^1^* and *sw^1^;dop^1^* and *Gl^1^ dop^1^* mutants. In the lower panels the advancing membrane front is highlighted during slow phase (yellow) and fast phase (white). The angle of the membrane front and the embryo surface is indicated. (G) The *sw^1^* and *Gl^1^* mutations both significantly enhance the defects in membrane growth during the slow phase of *dop^1^* homozygotes. *P<0.05, **P<0.001 (*t*-test); error bars indicate s.e.m.

We injected the apically localising mRNA *hairy* (*h*) into wild-type and *dop^1^* embryos before the fast phase of cellularisation and automatically tracked their movements (Fig. 7A). The net apical, i.e. minus end-directed, movement of mRNA particles was significantly reduced in the mutant embryos (Fig. 7B). This change was associated with decreases in the frequency, run lengths and velocity of apical particle transport in *dop* mutants (Fig. 7C-E). By contrast, the lengths and velocities of plus end runs of RNA particles were not significantly different between wild-type and *dop* mutant embryos (Fig. 7C,D). Qualitatively, the defects in apical *h* mRNA transport in *dop* mutants are similar to those exhibited by embryos in which components of the dynein-based mRNA transport machinery are compromised, including the RNA-binding protein Egalitarian, the adaptor protein Bicaudal D and the dynein co-factor Lissencephaly-1 (Bullock et al., 2006; Dix et al., 2013). These data provide direct evidence that Dop is important for normal dynein-dependent transport in the early embryo.

The requirement of Dop for dynein-dependent transport and normal phosphorylation of Dic suggested that the cellularisation defects in *dop* mutants might be related to a malfunction in dynein regulation. In contrast to *dop*, cytoplasmic dynein plays many important roles during oogenesis and early cleavage division (Gepner et al., 1996; Robinson et al., 1999). To test for a role of dynein transport in cellularisation we took advantage of genetic interactions of *dop* with *sw* and *Gl*. Since *sw^1^* and *Gl^1^* on their own did not affect cellularisation (data not shown), we examined whether *sw^1^* and *Gl^1^* would enhance the membrane growth defect of *dop^1^* mutants. Using brightfield video microscopy, we found that the defect in membrane growth in *dop^1^* mutants was strongly enhanced in both *sw^1^ dop^1^* and *Gl^1^ dop^1^* double mutants (Fig. 7F,G). In the double mutants, membrane growth was strongly compromised throughout cellularisation. These data implicate central players in dynein-mediated microtubule-based transport, Glued and Dic, in the regulation of membrane growth in cellularisation.

### Mutations in *dop* impinge on the distribution of Rab11 endosomes and the Golgi complex

**Fig. 8.**
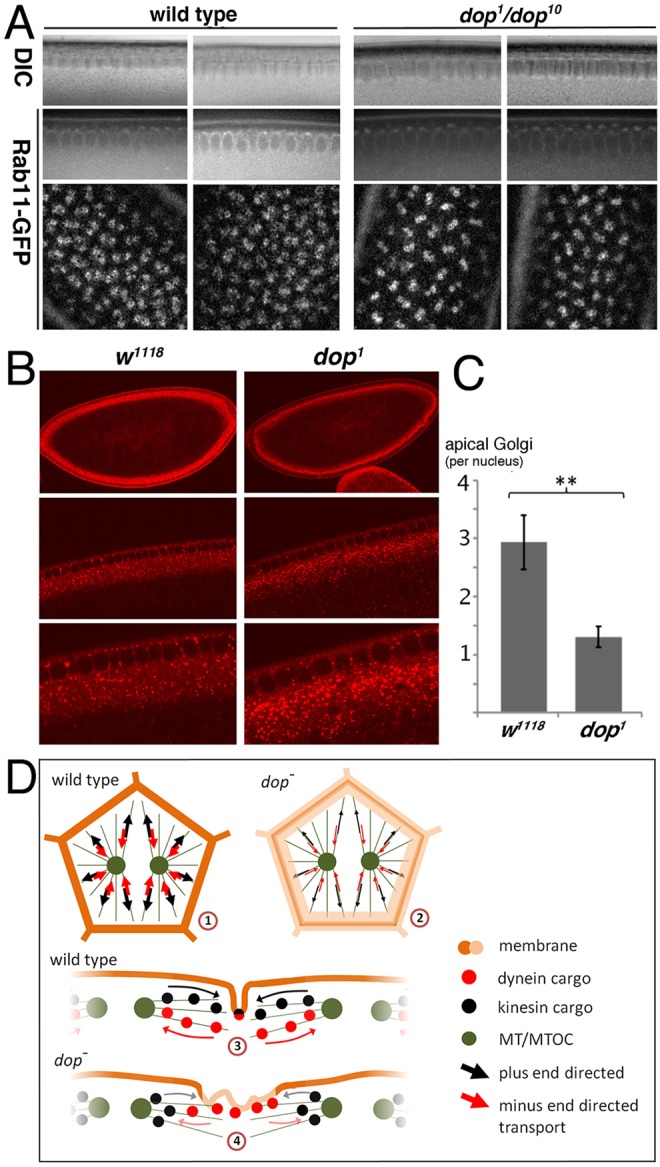
**Rab11 and Golgi compartments in *dop* mutant embryos.** (A) UAS-Rab11-GFP was expressed maternally (*mat67::Gal4*) in wild type and *dop^1^*/*dop^10^* transheterozygotes and imaged using a spinning disk microscope. DIC, Nomarski image. Left panels are at the beginning of slow phase and right panels are during slow phase. Top two rows show transverse sections, bottom row is a surface view. (B) Anti-p120 Golgi staining of *w^1118^* and *dop^1^* embryos at early slow phase stage. Representative embryos are shown for each genotype at increasing magnification (from top to bottom in each column). (C) Quantification of apical Golgi particles stained by anti-p120. There is a significant reduction in apical Golgi particles in *dop^1^* embryos as determined from transverse sections and normalised to the number of nuclei present on the section. **P<0.01 (*t*-test); error bars indicate s.e.m.; *w^1118^*, *n*=7; *dop^1^*, *n*=9. (D) Model of Dop function in cellularisation. (1) Transport along microtubules controls furrow formation; dynein and kinesin might regulate this transport in an interdependent fashion. (2) In the absence of *dop*, reduced Dic phosphorylation affects the efficacy of this transport in both directions. (3) In wild type, transport to and from the furrow region defines the lateral extent of the incipient furrow by deposition of F-actin, its regulators and endomembranes. (4) Lack of Dop results in an imbalance of dynein- and kinesin-mediated transport, interfering with the focussing of incipient furrows. MT, microtubules; MTOC, microtubule-organising centre.

Membrane growth in cellularisation requires both Golgi-derived vesicles and membrane supplied by the recycling endosome (Pelissier et al., 2003; Papoulas et al., 2005). Given the reduced membrane growth in *dop* mutants, we examined the localisation of the Golgi and the recycling endosome using antibodies and GFP-tagged Rab11 protein, respectively. During slow phase, Rab11-GFP exhibits dynamic localisation to, and in the vicinity of, the centrosomes apical to each nucleus (Fig. 8A; supplementary material Movies 9 and 10). In *dop* mutants, Rab11-GFP was more concentrated to the centrosomal area compared with controls (Fig. 8A; supplementary material Movies 11 and 12). We also analysed the distribution of a marker of the medial Golgi [p120, also known as Glg1 (Stanley et al., 1997)] in early cellularisation stages. Golgi membranes are transported in a dynein-dependent fashion to the apical cytoplasm in early cellularisation (Papoulas et al., 2005). We find that in *dop* mutants apical Golgi staining was reduced compared with control embryos (Fig. 8B,C). Together, these data indicate that *dop* is required for the normal distribution and transport of endomembrane systems that are crucial for membrane growth in cellularisation.

## DISCUSSION

To our knowledge, we present the first mutational analysis of a MAST kinase in any organism, and demonstrate that the MAST kinase Dop plays an important role in plasma membrane cortex compartmentalisation during the generation of epithelial polarity in the fly. The results reported here demonstrate a requirement of Dop in the establishment of the furrow canal and the bAJ at the cycle 14 transition. The defect in bAJ formation is likely to be a consequence of a failure in the initial specification of the incipient furrows. We propose that Dop acts upstream in furrow canal formation by controlling the formation of F-actin-rich foci, which initiate the assembly of a specific furrow membrane cortex.

In mid-cellularisation stages, *dop* mutant phenotypes are reminiscent of embryos lacking the early zygotic gene *bottleneck* (*bnk*) (Schejter and Wieschaus, 1993). In *bnk* mutants the initial formation of the cleavage furrows is normal, but then furrows close prematurely. Although we cannot exclude the possibility that *bnk* might play a role in later defects associated with *dop* mutations, the primary defect in *dop* mutants concerned the lack of regular F-actin-rich furrows during the onset of cellularisation. Another early zygotic gene, *nullo*, is required for the proper recruitment of F-actin during furrow canal formation (Sokac and Wieschaus, 2008b). Nullo and the actin regulator RhoGEF2 have been proposed to act in parallel pathways controlling processes that are distinct but both essential for F-actin network formation during the establishment of the furrow canal (Grosshans et al., 2005). Since early F-actin rearrangements are largely normal in *nullo* and *RhoGEF2* single mutants, we propose that Dop is essential for the initial early focussing of F-actin, whereas Nullo and RhoGEF2 are required to elaborate and maintain F-actin levels to stabilise the furrows. The actin regulator *enabled* (*ena*) has been shown to act downstream of Abelson tyrosine kinase (*Abl*) in the redistribution of F-actin from the plasma membrane cortex into the furrows in both syncytial stages and cellularisation (Grevengoed et al., 2003). Although *ena* would provide a good candidate for acting downstream of *dop* in the redistribution of F-actin, *ena* is already required for syncytial cleavages and the F-actin phenotypes in *Abl* mutants are much more severe than those that we found for *dop* mutants.

The similarity of syncytial cleavage furrows and the cleavage furrows at cellularisation raises the question of how they differ from each other. The molecular basis of the hexagonal pattern of the F-actin-rich cell cortex at the cleavage furrow relies upon the recycling endosome components Rab11 and Nuclear fallout (Nuf) (Riggs et al., 2003) and the actin polymerisation factors Dia (Afshar et al., 2000) and Scar/Arp2/3 (Zallen et al., 2002). In contrast to *dop* mutants, *nuf*, *dia* or *Scar* mutants indicate that these genes are required also for the dynamic redistribution of F-actin during syncytial development. Since Dop is a maternally supplied protein, its activity might be regulated by events triggered during the cycle 13-14 transition. The major difference between the furrows in syncytial stages and cellularisation is that metaphase furrows are formed during M phase, whereas cellularisation furrows are formed during G2 phase. Since Dop is a maternally supplied gene product, one would have to implicate regulation of Dop by zygotic factors to explain its phenotype at the cycle 13-14 transition. An alternative possibility is that Dop is regulated by phosphorylation or other post-translational modification through the cell cycle machinery and that, in the absence of Cdk1-dependent phosphorylation, its phosphorylation state is changed. We provide evidence that Dop is indeed differentially post-translationally modified during syncytial versus cellular blastoderm stages. We propose that such cell cycle-dependent regulation of Dop may be crucial in transforming syncytial cleavages into persistent cellularisation furrows. Furthermore, our data suggest that this transition could require Dop-dependent regulation of dynein-associated microtubule transport.

The mechanisms for the initial localisation of Baz and E-cadherin are still unclear but, interestingly, *dop* is required for the localisation of both proteins. At the cycle 14 transition, E-cadherin and Arm puncta are associated with apical membrane projections and the homophilic association of these cadherin puncta is strengthened by membrane flow and is dependent on actin (McGill et al., 2009). Baz function allows these puncta to become tightened into sAJs. Thus, Dop might affect the stabilisation of the weakly interacting puncta either through cortical actin organisation or membrane flow. In addition to this early requirement for Baz localisation, Dop is also involved in clearing Baz from the basal cytoplasm during late cellularisation. The mechanism that eventually clears Baz from the basal cytoplasm depends on dynein-based transport (Harris and Peifer, 2005). Therefore, Dop is required for dynein-based transport of different cargoes during cellularisation: lipid droplets, mRNA particles, Golgi and Baz. We propose that the main function of Dop in cellularisation is in regulating dynein-mediated transport of important cargos along microtubules (Fig. 8D).

This study presents the first evidence for regulation of dynein-mediated transport by a MAST family kinase. We show that Dop controls phospho-Dic levels in a direct or indirect manner. The data are consistent with a model in which the initiation of furrow formation involves dynein-dependent transport that is controlled by Dop (Fig. 8D). In support of a role in membrane formation, we find defects in the distribution of the recycling endosome and Golgi compartments in *dop* mutants. Interference with *Rab11* function causes similar defects in Slam distribution as those shown by *dop* mutants (Pelissier et al., 2003). Therefore, Dop might control the transport of endomembrane compartments, which drive membrane growth. In addition, F-actin redistribution plays a major role in membrane cortical compartmentalisation in the initial stages of cellularisation (Sokac and Wieschaus, 2008a, 2008b). The focussing of F-actin to incipient furrows might involve a dynein-dependent shift of actin regulators or existing actin filaments to the furrow. An attractive hypothesis is that the translocation of F-actin and/or its regulators is coupled to an endomembrane compartment that is transported via microtubules towards the incipient furrow canals. Future studies should aim to determine which dynein cargos contribute to furrow formation and how Dop regulates Dic phosphorylation at the molecular level.

## MATERIALS AND METHODS

### *Drosophila* strains and culture

Flies were kept on standard medium and embryos were collected on yeasted apple juice agar plates. *dop* mutant alleles were created by EMS mutagenesis and selected for non-complementation of the *dop^1^* allele (Galewsky and Schulz, 1992). The chromosomal deficiencies *Df(3L)EP3417MR15* and *Df(3L)EP1754MR20* were created by male recombination using *P[EP]Ago2^EP3417^* (Meyer et al., 2006). Fly lines were obtained from Bloomington *Drosophila* Stock Center (Indiana University, IN, USA) unless otherwise indicated: *UAS::rab11-GFP*, *ubi::E-Cad-GFP* (Oda and Tsukita, 2001), *UAS::Baz-GFP* (gift of A. Wodarz, Göttingen, Germany), *mat-α-tubulin67;mat-α-tubulin15* (gift from D. St Johnston, Cambridge, UK), *NGT::Gal4* (gift from P. Gergen, Stony Brook, NY, USA).

### Molecular biology

Transgenes were created using a *pUASP-attB* vector for site-directed integration into *M[vas-int.Dm]ZH-2A, M[3xP3-RFP.attP′]ZH-58A* flies. A *CG6498* cDNA was used as template to generate Dop constructs (primers are listed in supplementary material Table S1). For *dop^ΔDUF^* (lacking amino acids 341-703), *dop^ΔKinase^* (lacking amino acids 828-1181) and *dop^ΔPDZ^* (lacking amino acids 1512-1593) constructs, the appropriate coding sequences were amplified by PCR and cloned into *pUASP-attB*. Equal expression levels in transgenic flies were confirmed by western blot. For tagged constructs the stop codon was replaced with 3×HA tag or EGFP coding sequences.

### Genetic characterisation of *dop* alleles

To map the break points of the non-complementary chromosomal deletions *Df(3L)XG9*, *Df(3L)MR15* and *Df(3L)MR20*, 45 different genomic regions of 200-600 bp were amplified by PCR in triplicate. Template genomic DNA was prepared from homozygous embryos, which were identified by immunostaining against β-galactosidase expressed from balancer chromosomes. In the case of *Df(3L)XG9*, the breakpoint was determined by genomic sequencing.

### Immunohistochemistry and microscopy

Embryos were fixed in 4% formaldehyde in PBS/heptane or by heat fixation (Müller, 2008). Primary antibodies were used at the following dilutions: mouse anti-Dlg 4F3 (1:500; DSHB), rabbit anti-Slam (1:5000; gift of J. Großhans, Göttingen, Germany), rabbit anti-Baz (1:5000; gift of A. Wodarz, Göttingen, Germany), mouse anti-Arm 7A1 (1:50; DSHB), mouse anti-Nrt BP-106 (1:10; DSHB), mouse anti-Dic 74.1 (1:2000; Abcam, ab23905), mouse anti-p120 Golgi (1:500; Calbiochem, no longer available), rat anti-E-Cad DCAD2 (1:50; DSHB) and rabbit anti-Dop 1303 (1:50; see below). Fluorophor-conjugated secondary antibodies (Molecular Probes) were used at 1:250 and DAPI at 1 µg/ml. Tubulin and Rhodamine-Phalloidin staining of F-actin were performed as described (Tang et al., 2001). Specimens were mounted in Mowiol/DABCO, imaged on a Leica SP2 confocal microscope and processed using ImageJ, Photoshop (Adobe) or Volocity (PerkinElmer).

For transmission electron microscopy, samples were fixed by high-pressure freezing and processed by freeze-substitution as described (Grosshans et al., 2005). For scanning electron microscopy, embryos were staged under halocarbon oil, fixed and processed as described (Müller and Wieschaus, 1996). Specimens were dehydrated in a graded ethanol series and dried using tetramethylsilane and coated with 20 nm gold-palladium. Microscopy was performed on a Hitachi S4700 scanning electron microscope and on a JEOL 1200EX transmission electron microscope.

### Quantification of imaging data

All quantifications were made using raw data in ImageJ (Schneider et al., 2012). Membrane growth was measured from movies acquired by brightfield video microscopy. An area of 1×326 pixels was taken from each time point to be converted with the multiple kymograph plug-in (Seitz and Surrey, 2006). The angle between the invaginating membrane and vitelline membrane was used to indicate the speed of membrane growth. The circumference of eyes was tracked using the line tool and the area was measured using the multi-measure plug-in. Golgi particles positive for p120 immunolabelling were monitored from raw imaging data using the threshold tool (Photoshop). Particles apical to the nuclei were quantified from transverse optical sections.

### Live imaging and analysis of mRNA motility

GFP-expressing embryos were staged to cycle 10 in halocarbon oil using a dissection microscope. Selected embryos were manually de-chorionated and glued to a cover slip. Movement of E-cad-GFP or Baz-GFP was recorded on Leica SP2 or SP5 microscopes, with one image captured every 5 s. Transverse optical sections were taken in the mid-dorsal region and surface views were recorded from 8 µm stacks with a step size of 0.5 µm and displayed as maximum intensity projections. *Rab11-GFP* was recorded on a CSU-X1 system (Yokogawa) spinning disk microscope using a 60×/1.42 NA oil-immersion lens.

Alexa488-labelled capped RNA corresponding to the *h* 3′UTR was synthesised and injected into cycle 14 blastoderm embryos obtained from *dop^1^* females fertilised with *dop^1^* males or into wild-type embryos from an Oregon-R strain as described previously (Bullock et al., 2006). Images were captured every 297 ms using an Ultraview ERS spinning disk system (PerkinElmer) on an Olympus IX71 inverted microscope equipped with an Orca-ER camera (Hamamatsu) using a 60×/1.2 NA UPlanApo water objective. Automatic centroid-based tracking of mRNA particles was performed as described (Bullock et al., 2006).

### Protein analysis

Peptides from the N-terminus of Dop (ISTSTPQKNDEHQEQC and MSRQEGAASRPADGAC) were synthesised and used to immunise rabbits and to affinity purify antibodies (anti-Dop 1303) (Eurogentec). Protein extracts from staged embryos were prepared in lysis buffer (50 mM Tris-HCl pH 8.0, 150 mM NaCl, 1% Triton X-100, 0.1% β-mercaptoethanol) and run on 3-8% Tris acetate SDS-PAGE (Invitrogen) and transferred to PVDF membrane (Whatman, GE Healthcare) for immunoblotting. For 2D PAGE, protein extracts from visually staged embryos were acetone precipitated and redissolved in rehydration buffer [7 M urea, 2 M thiourea, 1.2% CHAPS, 0.25% ampholytes (GE Healthcare), 0.4% ASB-14, 43 mM DTT]. Protein concentration was established using 2-D Quant (GE Healthcare). Equal amounts of protein were separated on pH 4-7 Immobiline DryStrip gels (Amersham) with the IPGphor isoelectric focusing system (Amersham). The second dimension was performed on 4-12% bis-Tris Zoom gels (Invitrogen). Signal intensity on immunoblots was measured using ImageJ.

For dephosphorylation assays, 100 μg protein lysate was incubated with 5 µl λ-protein phosphatase (New England BioLabs) at 30°C for 30 min. As control, phosphatase was heat inactivated for 1 hour at 65°C in the presence of 50 mM EDTA.

## Supplementary Material

Supplementary Material
