# Supplementary material for: The Drosophila MAST kinase Drop out is required to initiate membrane compartmentalisation during cellularisation and regulates dynein-based transport
Source: Development. 2014 May;141(10):2119–30. doi: 10.1242/dev.104711 (PMC4011086; doi:10.1242/dev.104711)
Supplement: Supplementary Material [file supp_141_10_2119__index.html]

The Drosophila MAST kinase Drop out is required to initiate membrane compartmentalisation during cellularisation and regulates dynein-based transport — Supplementary Material 

# The *Drosophila* MAST kinase Drop out is required to initiate membrane compartmentalisation during cellularisation and regulates dynein-based transport

## DEV104711 Supplementary Material

**Files in this Data Supplement:**

- **Supplementary Material**
