# Supplementary material for: The Drosophila MAST kinase Drop out is required to initiate membrane compartmentalisation during cellularisation and regulates dynein-based transport
Source: Development. 2014 May;141(10):2119–30. doi: 10.1242/dev.104711 (PMC4011086; doi:10.1242/dev.104711)
Supplement: Supplementary Material [file supp_141.10.2119_DEV104711.pdf]

Supplementary Material

The Drosophila MAST kinase Drop out is required to initiate membrane compartmentalisation during cellularisation and regulates dynein-based transport

SUPPLEMENTARY MATERIAL

Daniel Hain[1], Alistair Langlands[1], Hannah C. Sonnenberg[1], Charlotte Bailey[1], Simon L. Bullock[2] and H.-Arno J. Müller[1]

1 Division of Cell and Developmental Biology, College of Life Sciences, University of Dundee, Scotland

2 Division of Cell Biology, MRC Laboratory of Molecular Biology, Francis Crick Avenue, Cambridge Biomedical Campus, Cambridge CB2 0QH, UK

Supplementary Material

Figure S1: Genetics of the *dop* locus.

The *dop* locus was genetically mapped to chromosome 3L in cytological band 71E1 (Meyer et al., 2006), but initially misidentified as an allele of *Argonaute 2* (Meyer et al., 2006; Hain et al., 2010). (**A**) The genomic breakpoints of non-complementing chromosomal deletions were determined to map *dop*. Break points of *Df(3L)XG9*, *Df(3L)MR15* and *Df(3L)MR20* are indicated by black bars. *MR15* and *MR20* were mapped by PCR only and breakpoints are located within the grey areas. *XG9* breakpoints have been determined by sequencing of genomic DNA as in 15524158 and 15659132, respectively. *Df(3L)XG9* is predicted to uncover the apparent lethal P-element insertion *P{lacW}l(3)s1754*, which resides at 15554702 and within the first exon of *CG7427*. Amplification of genomic DNA of the *CG7427* locus by PCR confirmed that *Df(3L)XG9* does uncover this gene. Since, as previously reported (Meyer et al., 2006) *P{lacW}l(3)s1754* does complement *Df(3L)XG9*, we conclude that the lethality associated with the *P{lacW}l(3)s1754* chromosome must be located elsewhere on the third chromosome. These results have been communicated to Flybase to correct the annotation of P{lacW}l(3)s1754 (http://flybase.org/reports/FBrf0222642.html). (**B**) Cartoon of the domain composition of *Drosophila* and human microtubule-associated Ser/Thr kinases (MAST).

Figure S2: Distribution of Dop-HA and GFP in syncytial divisions.

Dop-HA or Dop-GFP were expressed maternally using one copy of the *nos-Gal4* driver. (**A**) Dop-HA expressing embryos were fixed and immunostained with antibodies against HA (red) and for DNA using DAPI (blue). Slightly higher levels of Dop-HA immunostaining is associated with the mitotic spindle. (**B**) Still images of a time-lapse recording demonstrating the dynamic movement of Dop-GFP in syncytial blastoderm stages. Similar to Dop-HA, during M-phase, higher levels of Dop-GFP can be observed to associate with the area where the mitotic spindle is localised.

Supplementary Material

Figure S3: Failure in separation of Dlg and Slam positive cortical domains. Embryos at mid-cellularisation were fixed and stained with antibodies against the furrow canal marker SLAM (green), the lateral membrane marker Dlg (red) and DAPI for DNA (blue). In wild type, Dlg-positive membrane cortex is clearly separated and does not extent into the Slam-positive cortex (arrow heads). In embryos derived from *dop[1]* homozygous mothers, Dlg domain extents into and overlaps with the Slam-positive domain (arrow heads).

Figure S4: Normalised representation of Figure 2E. Optical transversal section of wild-type and dop^1 mutant embryos expressing Baz::GFP under control of mat-tub::Gal4 during cellularisation. The brightness of the wild type panel was increased to match saturation levels of the dop^1 panel. Arrows point out the position of the cellularisation front. Basal signal of Baz-GFP in dop^1 mutant embryos is retained after cellularisation completes. Numbers indicate the time (min:sec) after initiation of cellularisation. The scale bars are 10 μm long.

Figure S5: Tubulin localisation in wild-type and *dop* mutants.
Microtubules in wild type (A-D) and embryos derived from dop^1 (E-H) homozygous mothers. Embryos were fixed in 37% formaldehyde and stained with anti β-Tubulin antibody (red; DNA is shown by DAPI staining in blue). To avoid bias during immunolabelling procedures, embryos were treated all the same and wild type and mutants were stained in together in one tube. Wild type embryos were discriminated from *dop* mutant embryos by pre-staining with anti-Vasa antibodies, which labels the primordial germ cells (**I,J**; green). Embryos were recorded by confocal microscopy with identical settings. Progressive stages during cellularisation are shown (arrowheads indicate the position of the furrow canals): (**A,E**) syncytial blastoderm; (**B,F**) early cellularisation (beginning of slow phase); (**C,G**) mid cellularisation (end of slow phase). (**D,H**) late cellularisation (fast phase). (**A, E**) In syncytial stages wild-type and dop^1 embryos do not show any difference. (**B,F**) During the early cellularisation wild-type embryos show

microtubule bundles, which arranged in typical basket-like structures surrounding the nucleus and thin filaments reaching into the yolk (arrows in B). *dop*^{1} embryos (a severe example is shown in F) at a comparable stage show irregular arrangement of microtubule bundles (F). However, nuclei are surrounded by the basket-like microtubules arrays even if they detach from the membrane.
(**C,G**) At mid cellularisation wild type with *dop*^{1} embryos exhibit a similar pattern. *dop*^{1} embryos with lower amount of nuclear detachment as shown here exhibits very similar microtubule staining as the wild type.(**D,H**) At late cellularisation stages embryos in both classes show long microtubule bundles reaching into the basal cytoplasm.

Movie 1: Dop-GFP in syncytial divisions.

Dop-GFP was expressed maternally using *nos*-Gal4 and surface fluorescence in living embryos was measured using confocal microscopy. In S-phase Dop-GFP is cytoplasmic. During M-Phase Dop-GFP dynamically associated with a structure surrounding the mitotic spindle.

Movie 2: Dop-GFP in syncytial divisions and cellularisation.

Dop-GFP was expressed maternally using *nos*-Gal4 and fluorescence in living embryos was measured using confocal microscopy. The movie shows a transversal view of an embryo during mitotic cycle 12, 13 and the slow phase of cellularisation. Dop-GFP is dynamically associated with the area of the mitotic spindles. At the onset of cellularisation Dop-GFP is slightly enriched in the domain apical of the nuclei.

Movie 3: Baz-GFP wt and *dop*^{1}.

UAS:Baz-GFP expressed by mat15:Gal4 (left) and UAS:Baz-GFP expressed by embryos from flies of the genotype UAS:Baz-GFP/*mat67*;*dop*^{10}/*dop*^{1} (right). Imaging speed was 6 time points per min. Transversal sections of the embryos are shown.

Supplementary Material

Movie 4: E-cadherin GFP in wild type.

Ubiquitously expressed E-Cadherin-GFP in wild type genetic background. Imaging speed was 2 time points per minute with a stack size of 9 µm. The movie shows a maximum intensity projection of the z-data.

Movie 5: E-cadherin GFP in *dop*^{1} mutant.

Ubiquitously expressed E-Cadherin-GFP in embryo derived from *dop*^{1}/*dop*^{1} females. Imaging speed was 2 time points per min. with a stack size of 9 µm. The movie shows a maximum intensity projection of the z-data.

Movie 6: Baz-GFP wt and *dop*^{1}.

UAS:Baz-GFP expressed by mat15:Gal4 (left) and UAS:Baz-GFP expressed by embryos from flies of the genotype UAS:Baz-GFP/*mat67*;*dop*^{10}/*dop*^{1} (right). Imaging speed was 6 time points per min. Surface views are shown. For display 8 µm z-stacks were recorded and displayed as maximum intensity projection.

Movie 7: Movement of *hairy* mRNA particles in wild-type embryo.

Alexa488-labelled capped RNA corresponding to the *Drosophila hairy* 3'UTR was synthesised and injected into cycle 14 blastoderm embryos derived from Oregon R wild type strain. Images were captured every 297 msec using spinning disk microscopy.

Movie 8: Movement of *hairy* mRNA particles in *dop*^{1} mutant.

Alexa488-labelled capped RNA corresponding to the *Drosophila hairy* 3'UTR was synthesised and injected into cycle 14 blastoderm embryos derived from *dop*^{1} homozygous females. Images were captured every 297 msec using spinning disk microscopy.

Movie 9: Rab11-GFP in wild-type embryo at onset of cellularisation. For Movies S14 to S17 imaging conditions and all settings were identical. Imaging

speed was 12 time points per min at a total of 2 min recording, 25 time points in total. Single z-planes are shown. Exposure time was 200 ms. Every time point was taken by an average of 4.

Movie 10: Rab11-GFP in wild-type embryo during slow phase.
Note dynamics of Rab11 endosome membranes from the centrosomal area to the periphery.

Movie 11: Rab11-GFP in dop[1]/dop[10] embryo at onset of cellularisation.
Note high accumulation of Rab11 endosome membranes to the centrosomal area compared to wild type.

Movie 12: Rab11-GFP in dop[1]/dop[10] embryo during slow phase.
Note high accumulation of Rab11 endosome membranes to the centrosomal area compared to wild type.

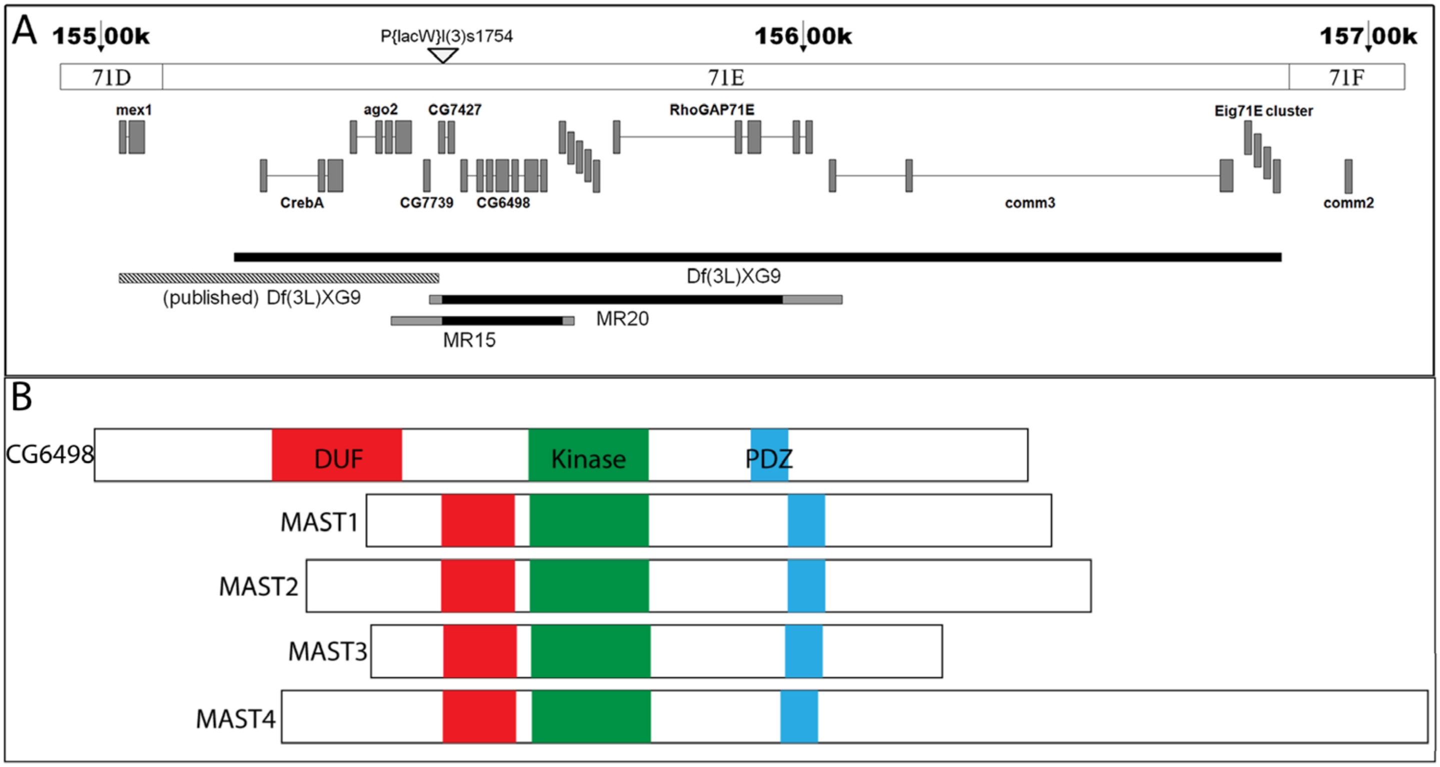

Figure S1

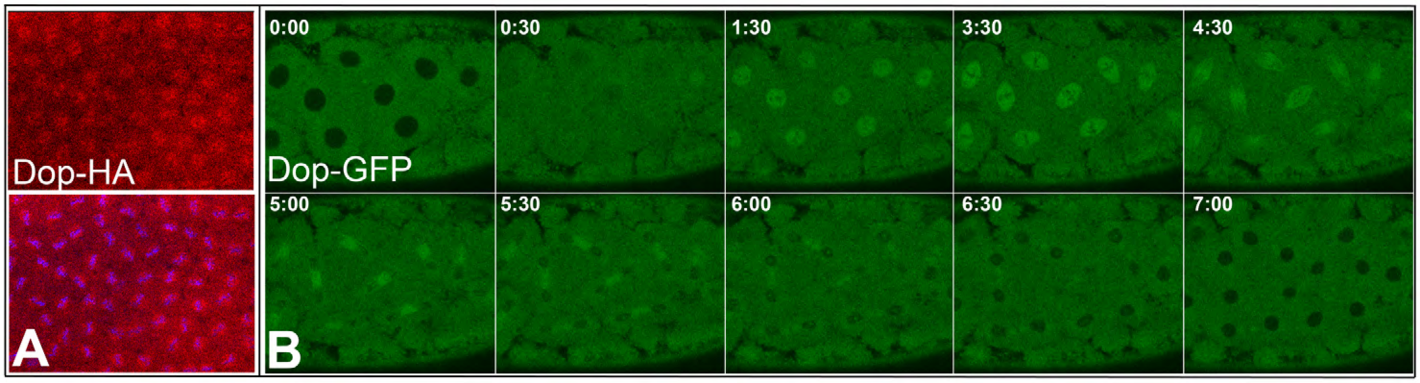

Figure S2

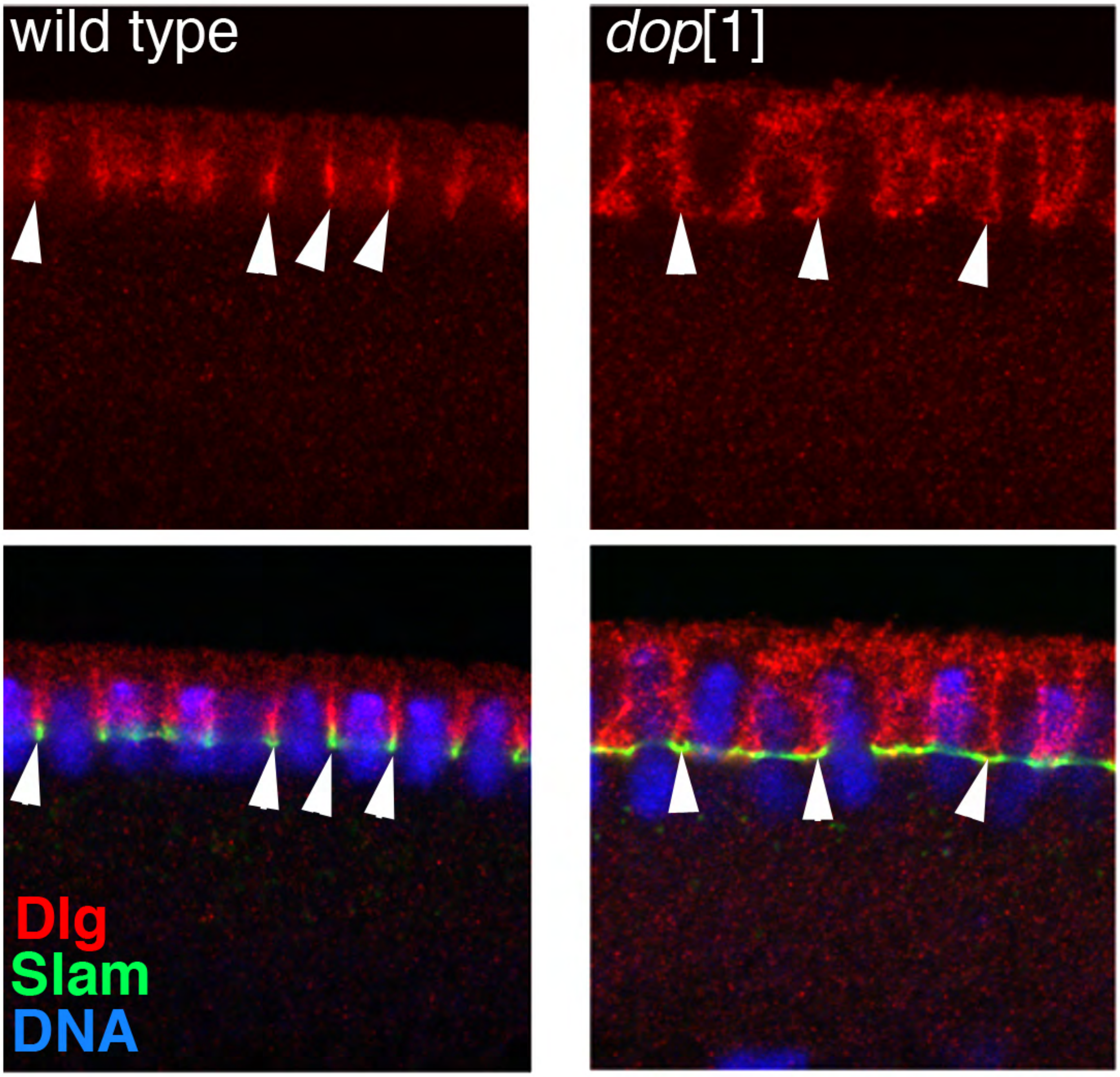

Figure S3

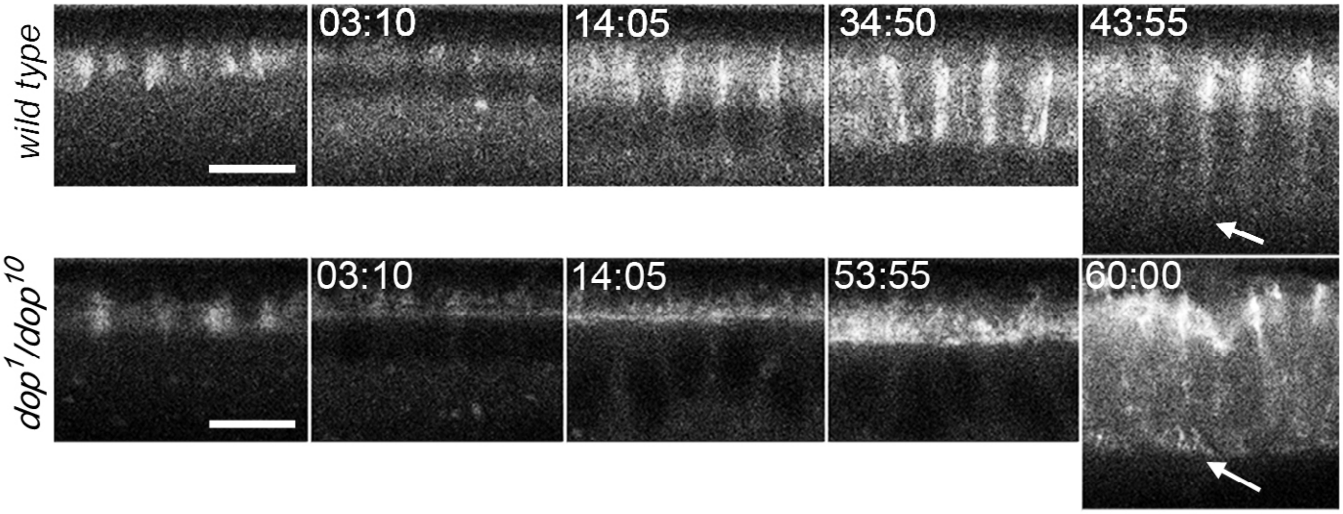

Figure S4

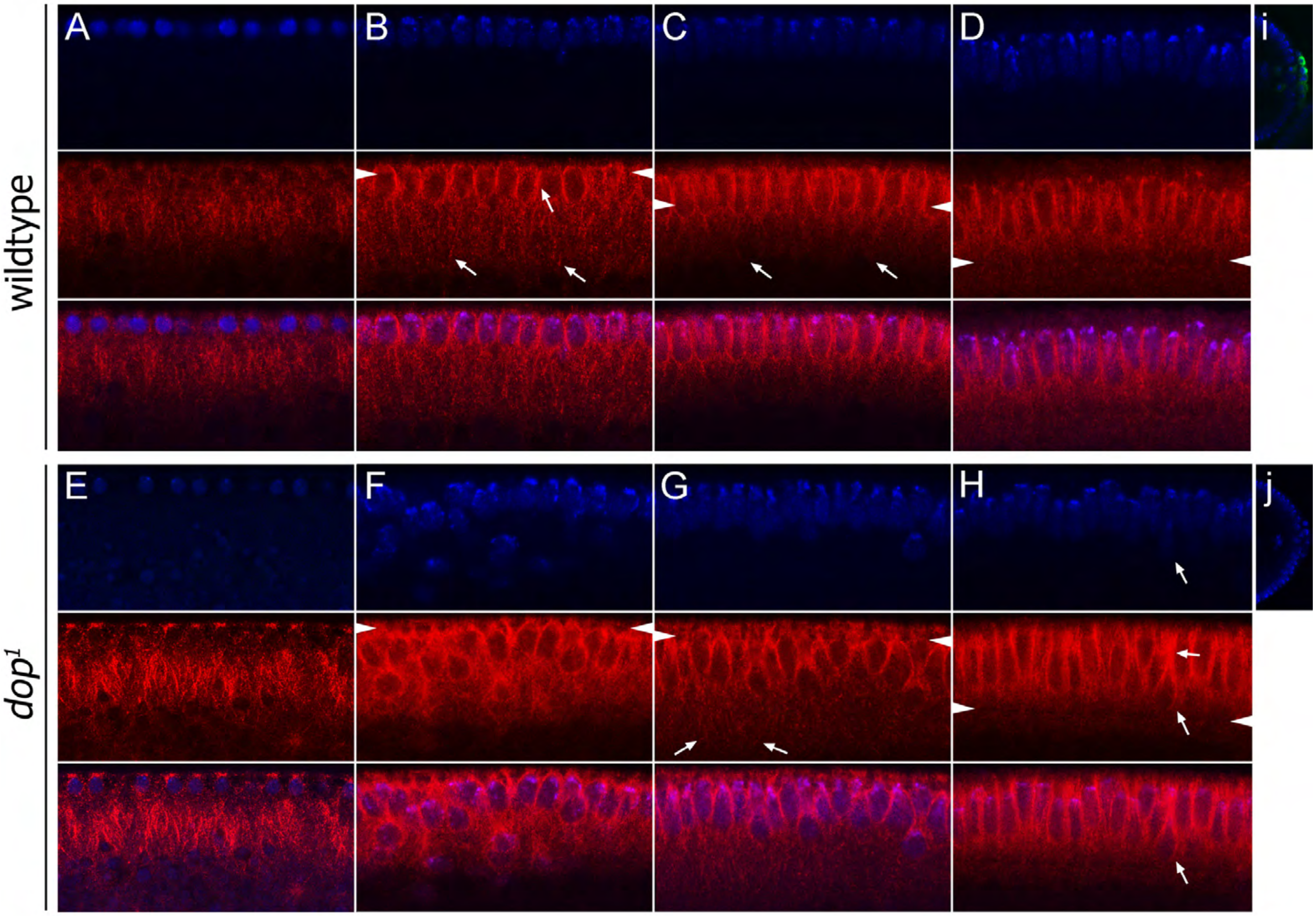

Figure S5

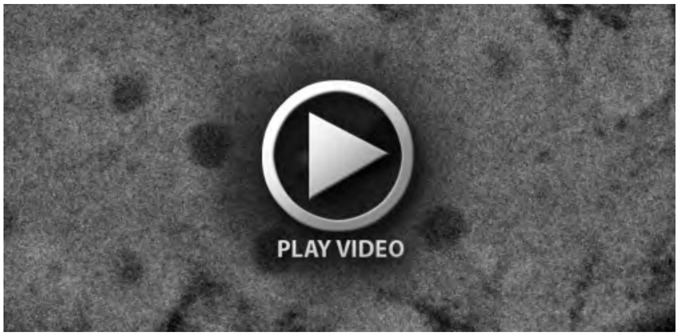

Movie 1.

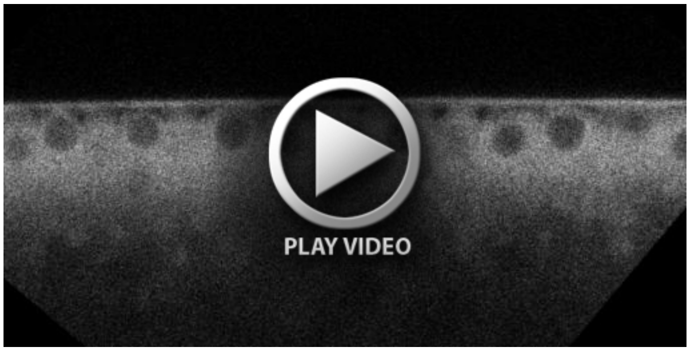

Movie 2.

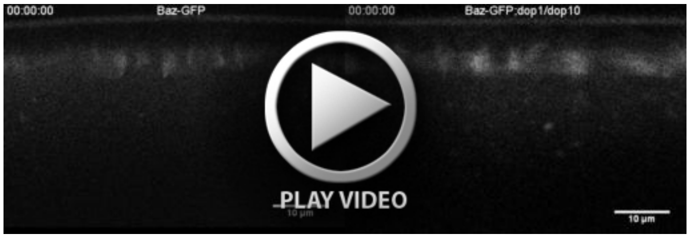

Movie 3.

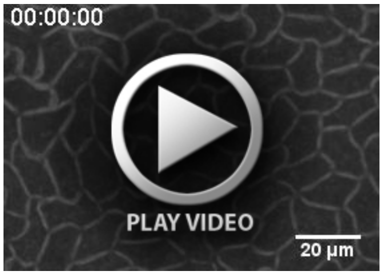

Movie 4.

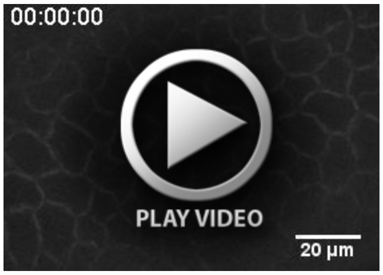

Movie 5.

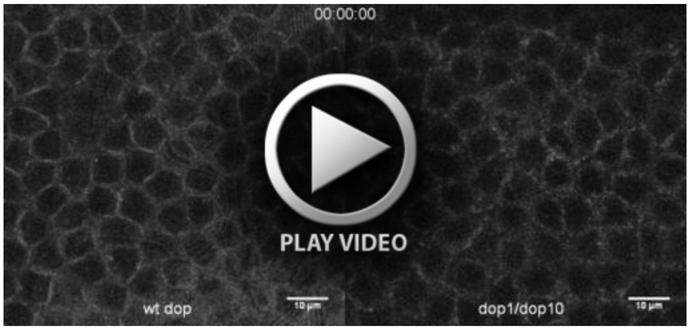

Movie 6.

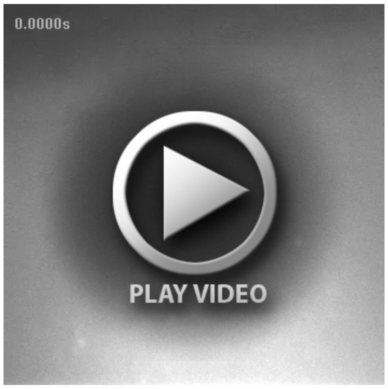

Movie 7.

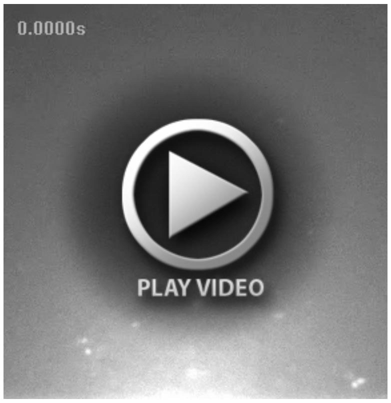

Movie 8.

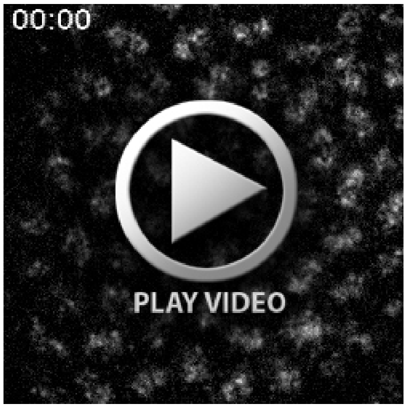

Movie 9.

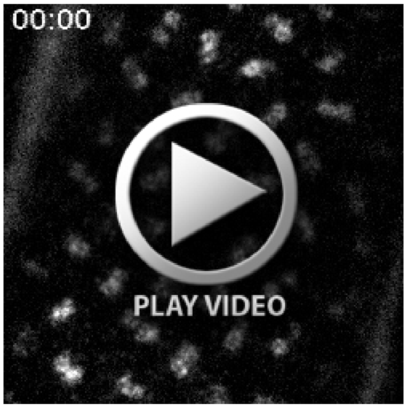

Movie 10.

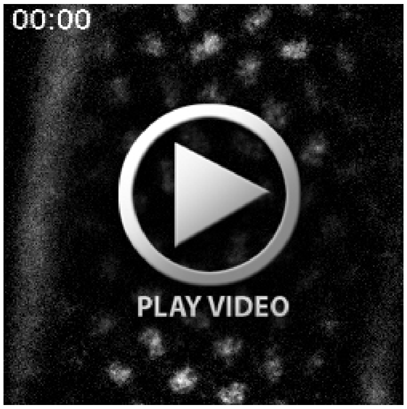

Movie 11.

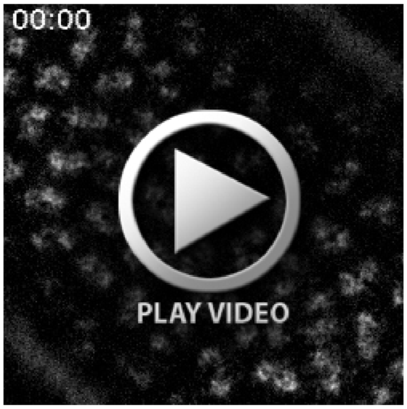

Movie 12.

Table S1. Primers

Primer Name	Sequence	TM
Cloning primers		
Dopfull NotI fwd	CA GCGGCCGC ATGAGTCGCCAGGAGGGAGC	75
Dopfull KpnI XbaI rev	CT TCTAGA GGTACC CTTCCTCTGCTTGGCAGCAGA	75
Delta Kinase 1 fwd	TAGGTACCATGAGTCGCCAGGAGGGAGCT	68
Delta Kinase 1 rev	TAGCGGCCGCGTTGTTGGGCTCCACTGCCAG	68
Delta Kinase 2 fwd	CTGCGGCCGCCATGCCACTCCCACGTCTACCG	72
Delta kinase 2 rev	GATCTAGACTTCCTCTGCTTGGCAGCAGAAG	72
dDUF2fwd	GCGGCCGCCAGCAGGAATTAAGGGAAACACAGC	74
dDUF1rev new	AC GCGGCCGCCCGAGTATGGGGAATGGGTTCG	70
dPDZ1fwd	GGTACCATGAGTCGCCAGGAGGGAGCTG	72
dPDZ1rev	GCGGCCGCCACGACGTATGATGATCGGAGGC	72
dPDZ2fwd	GCGGCCGCCACACCAGCATCCAGAGCGGTGG	78
dPDZ2rev	TCTAGACTTCCTCTGCTTGGCAGCAGAAGTG	78

Primer Name	Sequence
Sequencing primers	
CG6498_1fwd	GTGACCGTTCGGCTGATTGAT
CG6498_1 rev	GTTGCGATTACGGGGTTTTTG
CG6498_2 fwd	AGCGCCTTGCCAGCCGTATG
CG6498_2 rev	CTGCCCGACCGCGAAATGTT
CG6498_3 fwd	CAGCGGTACTTGATTTTCGGTTGG
CG6498_3 rev	TTTTGCGGCGTTGAGGTGGATA
CG6498_4 fwd	CGGGGTTCAGCTGGAGGAGGTG
CG6498_4 rev	TGGCGTTTGAGGAGGCTGTTGAGG
CG6498_5 fwd	CGAGGCGGCAGCTGAACTAAATG
CG6498_5 rev	AGGCGCTACCCGAACCACTACCAC
CG6498_6 fwd	TTTGCCGCGTTTCTCCATCTCG
CG6498_6 rev	ATTGCCCCCGCCTGCTATTACG
CG6498_7 fwd	CACGTCGCCCTCATCCTCTTC
CG6498_7 rev	CCGCGCCCATTCTCATCAC
CG6498_8 fwd	CCATCGCGTGCCCAGGAGACT
CG6498_8 rev	GAGATGGAGAAACGCGGCAAAATG
CG6498_9 fwd	CGTCCAACTTGCTGCGTATGC
CG6498_9 rev	CCGGGCGGAATGTTGTGC
CG6498_10 fwd	CTTTTCGCGCAGCACACCAGA
CG6498_10 rev	GCCGCAGTCCCGCCTCAA
